# AI/ML-Assisted SERS Biosensing for Biomolecular Detection: From Direct Spectral Response to Integrated Diagnostic Systems

**DOI:** 10.3390/bios16060346

**Published:** 2026-06-21

**Authors:** Jun Gyu Park, Woohyun Park, Suji Choi, Sanghyo Lee, Minseok Kim

**Affiliations:** 1 Semiconductor Specialized University Project Group, Kumoh National Institute of Technology, Gumi 39177, Republic of Korea; jungyu@kumoh.ac.kr (J.G.P.); pwh1011@kumoh.ac.kr (W.P.); suji983@kumoh.ac.kr (S.C.); 2 School of Materials Science and Engineering, Kumoh National Institute of Technology, Gumi 39177, Republic of Korea; 3 School of Mechanical Engineering, Kumoh National Institute of Technology, Gumi 39177, Republic of Korea; 4 Department of Aeronautics, Mechanical and Electronic Convergence Engineering, Kumoh National Institute of Technology, Gumi 39177, Republic of Korea

**Keywords:** surface-enhanced Raman scattering, SERS, biosensing, biomolecular detection, bio-recognition interface, signal transduction, digital SERS, artificial intelligence, machine learning, clinical translation

## Abstract

Surface-enhanced Raman scattering (SERS) offers a powerful route for biomolecular detection because it combines molecular specificity with high sensitivity, rapid optical readout, and multiplexing capability. In real biological samples, however, analytical performance is rarely determined by signal enhancement alone. Biofluids such as serum, plasma, saliva, urine, and interstitial fluid contain complex biomolecular mixtures that interfere with target capture, spectral response, and data interpretation. A practical SERS biosensor must therefore localize targets, stabilize spectral responses, tolerate matrix-induced variation, and convert complex spectra into reliable analytical information. This review discusses recent progress in SERS biosensing from an integrated system perspective, with particular focus on artificial intelligence/machine learning (AI/ML)-assisted interpretation. Direct label-free SERS provides chemically transparent readouts but is limited by stochastic adsorption, hotspot heterogeneity, and spectral variation in complex samples. Bio-recognition interfaces improve target localization, while signal-transduction strategies based on nanotags, immunoassays, clustered regularly interspaced short palindromic repeats (CRISPR) systems, nanozymes, and lateral-flow formats decouple molecular recognition from spectral generation. Digital SERS further improves measurement robustness by converting fluctuating intensities into countable, event-based outputs. AI/ML-assisted analysis can support full-spectrum classification, calibration transfer, explainability, and patient-level decision-making. We frame AI/ML-assisted SERS biosensing as an integrated architecture connecting substrate design, interface engineering, signal transduction, digital measurement, and clinical validation. Future progress will depend as much on validation-ready workflows as on plasmonic enhancement itself, especially for systems intended to operate across different samples, instruments, and clinical settings.

## 1. Introduction

Early and accurate biomolecular detection is central to modern diagnostics. Many clinically important targets, including cancer biomarkers, pathogen-derived nucleic acids, extracellular vesicles, inflammatory proteins, and metabolic indicators, are present at low abundance and must be measured in highly complex biological fluids. These samples are not simple chemical solutions. Serum, plasma, saliva, urine, and interstitial fluid contain dense and variable mixtures of proteins, salts, lipids, metabolites, vesicles, and cellular debris. Such components can compete for sensing surfaces, obscure weak molecular signals, and introduce large sample-to-sample variation [1,2,3,4]. The central challenge is therefore not only to detect a target, but to preserve analytical meaning under realistic biological conditions.

SERS is attractive for this task because it combines molecular specificity with optical sensitivity. By amplifying Raman scattering near plasmonic nanostructures, SERS can generate rich vibrational spectra from small sample volumes and short acquisition times. These features make it appealing for liquid biopsy, infectious disease testing, point-of-care diagnostics, and multiplexed biomarker analysis [5,6,7]. Much of the field has therefore focused on designing substrates that produce stronger electromagnetic hotspots [8,9,10,11,12]. Although this focus has been productive, hotspot engineering alone addresses only part of the biosensing problem.

A strong hotspot is only one part of the measurement. For a SERS signal to be useful, the target must reach the enhancing field, remain within an effective sensing distance, and produce a spectral response that is stable across spots, substrates, and samples. The resulting spectrum must also be interpreted correctly in the presence of matrix background and biological variability. These requirements explain why many highly sensitive SERS demonstrations remain difficult to translate into robust diagnostic workflows. In practical biosensing, the bottleneck has shifted from signal enhancement alone to the broader problem of localization, response generation, measurement stability, and interpretation.

Recent progress in SERS biosensing can be understood as a response to this problem. Direct label-free SERS remains the most chemically transparent strategy because the target molecule itself generates the measured spectrum. However, it is vulnerable to stochastic adsorption, molecular orientation effects, hotspot heterogeneity, and partial sampling of large biomolecules. Bio-recognition interfaces, including antibodies, aptamers, peptides, and molecularly imprinted polymers (MIPs), address this limitation by improving target localization and selectivity at the sensing surface [13,14,15,16,17,18]. Signal-transduction and amplification strategies go further by separating molecular recognition from spectral generation. In these systems, the target triggers a more measurable response through Raman nanotags, immunoassay structures, CRISPR-mediated reactions, nanozyme catalysis, or lateral-flow accumulation [19,20,21,22,23,24,25,26,27,28,29,30,31,32].

A further transition is now occurring at the level of measurement and interpretation. Digital SERS changes how signals are represented by converting unstable intensity-based responses into countable events, partitioned measurements, or binary-positive outputs [33,34,35,36,37]. In parallel, AI/ML-assisted analysis changes how spectra are interpreted [38,39,40,41,42]. Instead of relying only on selected Raman peaks, AI/ML methods can use full-spectrum patterns to classify samples, correct calibration differences, extract hidden spectral features, and support patient-level decision-making. This is particularly relevant in biofluid analysis, where disease-associated information is often expressed as small changes in the overall spectral profile rather than as a single new Raman peak.

The role of computational analysis should nevertheless be defined carefully. It cannot compensate for every weakness in the measurement. If spectra mainly reflect substrate defects, fouling, or batch-specific variation, a model may learn those artifacts instead of the biology of interest. The value of AI-assisted SERS emerges when the sensing architecture generates spectra that are reproducible, traceable, and sufficiently structured for model-based interpretation. In this sense, AI is not simply a final data-processing step. It is part of a broader design framework that affects substrate quality control, spectral acquisition, preprocessing, calibration transfer, validation strategy, and clinical decision-making.

**Figure 1:**
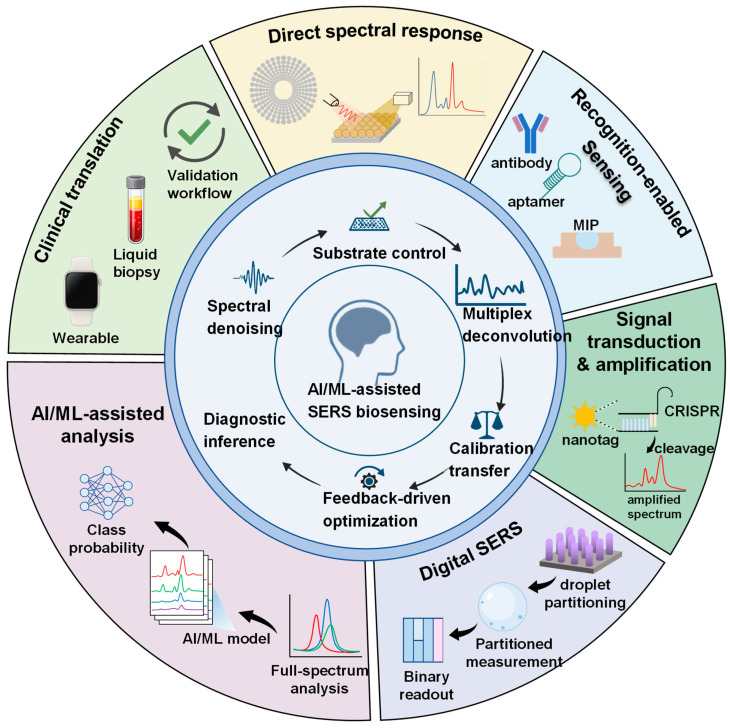
Conceptual framework of AI/ML-assisted SERS biosensing. The central wheel summarizes the AI/ML capabilities relevant to SERS—substrate quality control, multiplex deconvolution, calibration transfer, feedback-driven optimization, diagnostic inference, and spectral denoising—surrounded by the six thematic domains that organize this review: direct spectral response, recognition-enabled sensing, signal transduction and amplification, digital SERS, AI/ML-assisted analysis, and clinical translation.

In this review, we organize SERS biosensing around this architectural progression as shown in Figure 1. Rather than reviewing the field only by analyte type, substrate material, or biomedical application, we examine how successive layers of control have been introduced to improve analytical reliability. We first discuss direct label-free SERS as the foundational form of biomolecular spectral interrogation. We then examine bio-recognition interfaces that improve target localization, followed by signal-transduction and amplification strategies that produce more stable and measurable outputs. We next focus on digital SERS and AI/ML-assisted interpretation as key steps toward integrated diagnostic systems. Finally, we discuss analytical performance, biofluid validation, standardization, and regulatory considerations that will determine whether AI-assisted SERS biosensors can move from promising demonstrations to clinically actionable tools.

## 2. Direct Label-Free SERS and Substrate-Controlled Spectral Readout

Direct label-free SERS represents the most chemically transparent form of SERS biosensing. In this format, the target molecule itself generates the measured Raman spectrum after interacting with the plasmonic near-field. No Raman reporter, secondary label, enzymatic reaction, or nucleic-acid amplification step is required. The attraction is clear: if the analyte can be brought into an active hotspot, SERS can provide a rapid molecular fingerprint from a small sample volume.

The same directness also defines its main limitation. The measured signal is not determined by analyte concentration alone. It depends on whether the target reaches the hotspot, how it is oriented relative to the local field, which molecular groups are closest to the metal surface, and whether the resulting spectrum can be separated from the surrounding biological background [3,4,9,11]. In complex samples, these conditions are difficult to control. A spectrum-only readout can therefore be chemically rich but analytically unstable.

**Figure 2:**
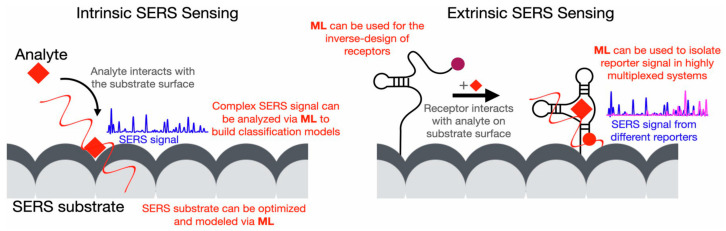
Direct spectral response and AI-assisted interpretation. The schematic contrasts intrinsic SERS sensing (**left**), where the analyte interacts directly with the substrate surface and ML is applied to optimize the substrate and classify complex spectra, with extrinsic SERS sensing (**right**), where a reporter-conjugated receptor binds the analyte at the surface and ML supports inverse receptor design and signal isolation in highly multiplexed systems. The different-colored spectral lines represent SERS signals from different Raman reporters. Adapted from Ref. [44] under CC BY 4.0.

Figure 2 frames this problem at the level of spectral interpretation. Direct SERS produces high-dimensional vibrational information, but diagnostically useful variation is often distributed across weak and overlapping spectral features rather than isolated Raman peaks. Conventional peak-height analysis may be sufficient for simple targets, but it becomes fragile when spectra arise from heterogeneous biofluids, extracellular vesicles, cells, or clinical samples. This is where data-driven analysis becomes useful [38,39,41,42,43]. Instead of forcing interpretation onto a few selected peaks, the full spectrum can be analyzed as a pattern that reflects the combined biochemical state of the sample. 

Before interpretation can be reliable, however, the physical measurement must be controlled. This is why substrate engineering remains central to label-free SERS. Colloidal Au and Ag nanoparticles can generate intense hotspots through aggregation, but their local geometry is difficult to reproduce [11,45,46]. Self-assembled or liquid-state plasmonic arrays improve mesoscale order and can provide more uniform measurement environments [11,47,48]. Lithographically defined structures improve spatial reproducibility further. Gap-mode systems, including nanoparticle-on-mirror structures and DNA-origami-templated dimers, offer even tighter control over nanogap geometry and analyte access [49,50,51].

**Figure 3:**
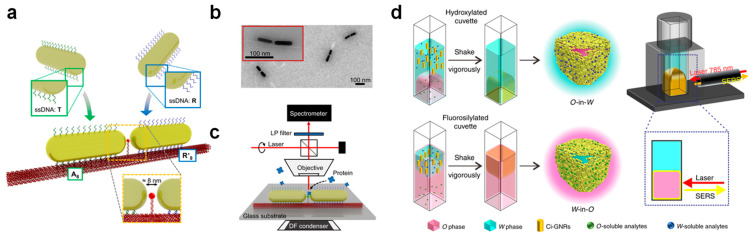
Plasmonic substrate engineering for more reliable SERS measurements. (**a**) DNA-origami–templated assembly of gold nanorod dimers with tip-to-tip alignment and a ~8 nm gap, providing accessible hotspots for single-protein SERS. (**b**) TEM characterization of the assembled dimers (scale bars: 100 nm). (**c**) Dark-field SERS optical setup for single-protein measurement using DF condenser, objective, LP filter and spectrometer. (**d**) Liquid-state quantitative SERS analyzer based on self-ordered metallic liquid–like plasmonic arrays in hydroxylated and fluorosilylated cuvettes, generating O-in-W and W-in-O assemblies of citrate-capped gold nanorods (Ci-GNRs) for 785 nm SERS interrogation of O- and W-soluble analytes. Panels (**a**–**c**) adapted from Ref. [51] under CC BY 4.0. Panel (**d**) adapted from Ref. [48] under CC BY 4.0.

Figure 3 illustrates this progression using two representative substrate-engineering strategies. DNA-origami-templated gold nanorod dimers represent a molecular-precision approach, in which plasmonic gaps are defined by a programmable scaffold to improve hotspot accessibility and structural reproducibility [51]. In contrast, self-assembled liquid-state plasmonic arrays provide a more scalable route toward spatially averaged and internally referenced quantitative SERS [48]. These two examples highlight a recurring trade-off in substrate design: molecular-level control can improve hotspot definition, whereas larger-area assemblies can improve practical measurement uniformity and throughput.

In addition to enhancement magnitude, substrate morphology should be considered as a data-quality variable in AI/ML-assisted SERS. Nanoparticle size, shape, gap distribution, surface roughness, aggregation state, and spatial hotspot density can all affect the measured spectrum. These morphological factors influence not only signal intensity but also spectral reproducibility, mapping heterogeneity, and batch-to-batch variation. Therefore, a SERS dataset collected from substrates with uncontrolled morphology may contain spectral patterns that reflect substrate variation rather than biomolecular differences. This issue becomes especially important when AI/ML models are trained on spectra collected from different substrate batches. If substrate morphology differs systematically between experimental groups, the model may learn morphology-related spectral variance instead of target-related biological information. For this reason, future AI/ML-assisted SERS studies should report substrate morphology, batch information, mapping conditions, and quality-control criteria together with model performance.

Even with improved substrates, label-free SERS remains vulnerable to interfacial variability. Small molecules may fit within the effective hotspot volume and generate relatively clear fingerprints. Larger biomolecules behave differently. Proteins, extracellular vesicles, pathogens, and cells often exceed the spatial range of the plasmonic near-field, so the measured spectrum may reflect only the surface-exposed portion of the target rather than the whole biomolecular structure [43,52,53]. In this case, SERS does not capture a complete molecular fingerprint. It captures a partial interfacial projection of the target under a specific adsorption geometry.

The problem becomes more severe in biological fluids. Serum, plasma, saliva, urine, and interstitial fluid contain abundant proteins, metabolites, salts, lipids, vesicles, and other matrix components that compete for plasmonic surfaces. These species can block hotspots, alter adsorption geometry, change aggregation behavior, and introduce background bands. For direct label-free SERS, matrix interference is not merely noise added after signal generation. It directly changes the sensing interface.

Direct label-free SERS therefore plays two complementary roles in modern biosensing. First, it remains a powerful method for molecular fingerprinting when the analyte and substrate environment are well controlled. Second, it provides a data-rich profiling approach for complex biological samples, where the goal is often not to assign every band to a single molecule but to classify a reproducible spectral pattern associated with a biological state [54]. The second role is where AI/ML becomes especially relevant.

Careful measurement therefore remains essential. Indeed, label-free SERS requires especially strict control of substrate quality, acquisition conditions, and preprocessing because the model has access to every variation present in the spectra. If spectra are dominated by substrate defects, uncontrolled aggregation, or batch-specific artifacts, machine learning may classify experimental noise rather than biological information [55,56,57]. The value of AI-assisted label-free SERS therefore depends on the same physical requirements that motivated substrate engineering in the first place: reproducible hotspots, controlled acquisition, stable preprocessing, and traceable sample metadata.

Direct label-free SERS should therefore be viewed as the foundation of AI-assisted SERS biosensing, not as a complete solution. It establishes the direct link between biomolecules and vibrational spectra. At the same time, it reveals why additional layers of control are needed. Bio-recognition interfaces address target localization. Signal-transduction strategies stabilize spectral generation. Digital SERS regularizes measurement events. AI/ML-assisted analysis then converts structured spectral variation into diagnostic information. This progression forms the basis of the following sections.

## 3. Recognition-Enabled SERS: From Random Adsorption to Controlled Target Localization

Direct label-free SERS relies on spontaneous interaction between the analyte and the plasmonic surface. This is powerful when the target is small, abundant, and chemically compatible with the metal interface. In most biosensing problems, however, these conditions are not guaranteed. Clinically relevant targets are often dilute, embedded in complex matrices, and unable to occupy hotspots reproducibly. Recognition-enabled SERS emerged to address this problem. Its primary role is not simply to improve selectivity, but to convert random adsorption into controlled target localization.

In SERS, binding alone does not guarantee a measurable signal. A recognition layer is often described as a biochemical filter that separates the target from background molecules. In SERS, it has an additional physical function: it determines where the target sits relative to the plasmonic near-field. Even a highly specific receptor may fail analytically if it places the target too far from the hotspot or creates a thick, fouling-prone interface. For this reason, bio-recognition in SERS should be viewed as interface engineering rather than affinity chemistry alone.

Antibody-functionalized SERS platforms remain one of the most widely used examples of recognition-enabled sensing, especially when the target is a clinically established protein biomarker [13,20]. Recent multiplexed immuno-SERS formats further extend this principle by encoding several biomarker-related signals with distinct Raman reporters, as demonstrated in multiple PSA-mediated PHI assays [19]. Antibodies provide high affinity, broad biomedical relevance, and compatibility with clinical immunoassay formats. They are particularly useful in sandwich-type SERS assays, where a capture antibody immobilizes the biomarker and a Raman-tagged detection probe generates the optical signal. However, antibodies are large. Their size introduces a distance penalty between the captured target and the plasmonic surface, and random immobilization can block binding domains or produce heterogeneous surface coverage. The performance of antibody-SERS platforms therefore depends on orientation control, probe density, spacer design, and antifouling backfilling as much as on binding affinity itself [58,59,60,61,62,63,64].

Aptamers and peptides offer a more compact alternative. Aptamers can be chemically synthesized, modified with defined linkers, and designed to undergo target-induced conformational changes [15,65,66,67]. This makes them useful when the sensing mechanism depends on changing the distance between a reporter, target, and hotspot. Peptides provide even shorter recognition motifs and can reduce steric crowding at the interface [16]. These properties are attractive for SERS because near-field coupling decays rapidly with distance. In practical terms, smaller recognition elements can improve the probability [15,65,66,67] that a binding event produces a measurable spectral response.

**Figure 4:**
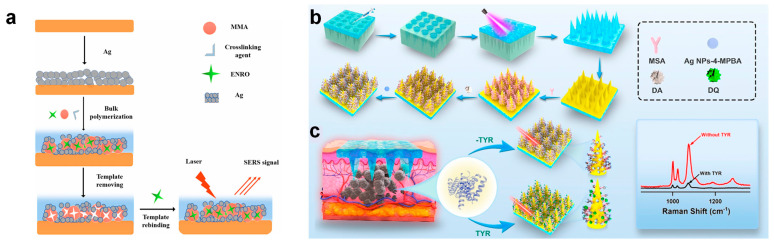
Recognition-enabled SERS interfaces for controlled target localization. (**a**) Molecularly imprinted polymer (MIP)–SERS sensor built on a Ag NP film, where bulk polymerization with MMA, crosslinking agent and enrofloxacin (ENRO) template forms selective rebinding cavities for label-free SERS detection of antibiotic residues in food. (**b**) Fabrication route for an Au-coated microneedle SERS biosensor, sequentially functionalized with mercaptosuccinic acid (MSA), Ag NPs–4-MPBA, dopamine (DA) and dopaminequinone (DQ) for in situ skin sampling. (**c**) In situ tyrosinase (TYR) sensing in skin: the microneedle SERS substrate distinguishes tyrosinase-positive from tyrosinase-negative skin via characteristic Raman shifts. Panel (**a**) adapted from Ref. [68] under CC BY 4.0. Panels (**b**,**c**) adapted from Ref. [69] under CC BY 4.0.

MIPs provide a different route to recognition. Instead of using a biological receptor, MIP-SERS platforms create shape- and chemistry-complementary cavities in a synthetic polymer matrix. This approach offers several advantages: improved storage stability, resistance to harsh conditions, and lower dependence on biological probe integrity. Figure 4 illustrates this principle through a MIP-SERS platform designed for selective analyte capture [68]. In this case, the imprinted cavity functions as a synthetic recognition site that confines the analyte near the SERS-active substrate, allowing selective spectral readout without relying on antibodies or aptamers.

The strength of MIP-SERS is also its main design challenge. Recognition occurs inside a polymeric matrix, and that matrix can become a barrier if it is too thick, too dense, or poorly aligned with the plasmonic field. A target captured outside the effective near-field may be recognized chemically but remain invisible spectroscopically. For MIP-based SERS, performance therefore depends on coordinating cavity position, matrix thickness, molecular diffusion, and plasmonic coupling. This makes MIP-SERS a clear example of why recognition and signal generation cannot be designed separately.

Recognition-enabled SERS is also moving beyond planar substrates and conventional assays. Microneedle-integrated SERS patches, such as the tyrosinase sensing platform shown in Figure 4, extend recognition into wearable and in situ formats [69]. Here, the sensing process is not simply target capture on a flat surface. The device must penetrate or contact biological tissue, sample a local biochemical environment, and generate a Raman-readable response under practical constraints. Recognition-enabled sensing therefore expands beyond surface capture: the recognition event is embedded in a device architecture that controls sampling, transport, and optical readout at the same time.

Recognition interfaces also influence the quality of downstream data analysis. By narrowing the range of adsorption geometries and suppressing nonspecific background, they can make spectral patterns more reproducible before any computational model is applied. A poorly controlled interface produces broad variability in adsorption geometry, background signal, and intensity [55]. A model trained on such data may learn surface artifacts rather than target biology. A well-designed recognition interface narrows that variability before computation begins.

This does not mean that recognition layers solve the SERS biosensing problem completely. Antibodies may improve specificity but increase distance from the hotspot. Aptamers and peptides reduce steric penalties but may be sensitive to ionic strength or matrix composition. MIPs provide robustness but can introduce diffusion and thickness limitations. Wearable recognition platforms improve deployment potential but add mechanical and sampling variability. In every case, the central question remains the same: does recognition produce a stable and interpretable SERS response.

Recognition-enabled SERS therefore represents the first major step beyond direct label-free interrogation. It gives the sensing system control over target localization. However, many captured targets still do not generate strong or stable Raman signals on their own. This limitation motivates the next architectural layer: signal transduction and amplification. Rather than asking the target itself to produce the measured spectrum, transduction-based systems convert the recognition event into a stronger, more stable, and more measurable SERS output.

## 4. Signal Transduction and Amplification: From Target Spectrum to Designed Response

Bio-recognition interfaces improve where the target is captured. They do not always solve what is measured. Many clinically relevant targets are weak Raman scatterers, present at low abundance, or difficult to place reproducibly inside a hotspot. Even when capture is selective, the intrinsic Raman signal of the target may remain weak, variable, or buried under the biological background. Signal-transduction strategies were developed to address this problem.

Conceptually simple as it is, this shift substantially changes the sensing architecture. In transduction-based SERS, the target does not need to generate the measured spectrum by itself. Instead, the target triggers a recognition event, and that event is converted into a stronger or more structured Raman response. This changes the role of SERS. It is no longer only a method for directly reading the target molecule. It becomes a method for reading an engineered assay output.

Reporter-mediated nanotags are the clearest example of this shift. A Raman reporter molecule is placed on or inside a plasmonic nanoparticle, often with a protective shell and a targeting ligand [13,19,20,70]. The reporter is chosen because it produces a strong, stable, and spectrally distinct signal. The biological recognition element is chosen for specificity. In this architecture, recognition and spectral generation are no longer the same process. The target determines where the nanotag binds, but the reporter determines what spectrum is measured.

This separation is useful for several reasons. First, it reduces dependence on the intrinsic Raman cross-section of the biomarker. A protein, nucleic acid, vesicle, or pathogen does not need to be a good Raman scatterer if it can recruit a strong SERS tag. Second, it improves multiplexing. Different reporters can generate distinct Raman peaks, allowing several targets to be measured in parallel [13,19,23]. Third, it makes the output more compatible with quantitative and AI-assisted analysis because the spectral features are predefined rather than entirely dependent on uncontrolled adsorption geometry.

Immuno-SERS extends this logic into a familiar diagnostic format. In a typical sandwich immuno-SERS assay, a capture antibody immobilizes the target, and a Raman-tagged detection antibody generates the readout [13,19,71]. The measured spectrum is therefore produced by the tag, not by the antigen itself. This architecture is especially useful for protein biomarkers, where direct label-free SERS is often limited by orientation effects and surface-biased sampling. However, immuno-SERS also introduces new design constraints. The capture surface, target, antibody layer, reporter particle, and plasmonic hotspot must be positioned in a compatible geometry. Too much spacing weakens the signal. Too much probe density increases steric hindrance and nonspecific binding. In this sense, immuno-SERS is not simply an immunoassay with Raman detection; it is a layered signal-assembly architecture.

An important nuance is that the need for AI/ML is not the same in all immuno-SERS formats. In a simple single-target sandwich immuno-SERS assay, the measured spectrum is generated mainly by a known Raman reporter rather than by the antigen itself [13,19,20,21]. If the reporter peak is strong, isolated, and reproducible, conventional peak-intensity analysis, ratiometric calibration, or classical multivariate analysis may be sufficient [13,19,31]. In this case, AI/ML is not essential for identifying the target signal, because the spectral channel has already been defined by the reporter chemistry.

However, the role of AI/ML becomes more important as the immuno-SERS assay becomes more complex. In highly multiplexed nanotag assays, reporter peaks can overlap, reporter loading can vary among particle batches, and matrix background can distort the apparent intensity of the tag signal [20,22,23,31,44]. In lateral-flow or imaging-type immuno-SERS formats, the measured response can also depend on test-line heterogeneity, reader alignment, and spatial sampling position. Under these conditions, AI/ML is useful not because the reporter spectrum is unknown, but because the assay output must be deconvoluted, normalized, quality-controlled, and transferred across batches, instruments, or sample matrices [44,72,73,74]. Thus, AI/ML should be considered optional in robust singleplex immuno-SERS, but increasingly valuable in multiplexed, spatially heterogeneous, or clinically deployed immuno-SERS systems.

Recent multiplexed immuno-SERS systems show how this reporter-mediated architecture can be extended from single-biomarker detection to multicomponent clinical assessment. For example, multiple PSA-related biomarkers can be encoded with different Raman reporters and assembled into a satellite immuno-nanocomplex, allowing for PHI-guided prostate cancer screening from clinical samples [19]. This type of assay illustrates why reporter-based SERS is useful not only for signal amplification, but also for structured and multiplexed diagnostic readout.

**Figure 5:**
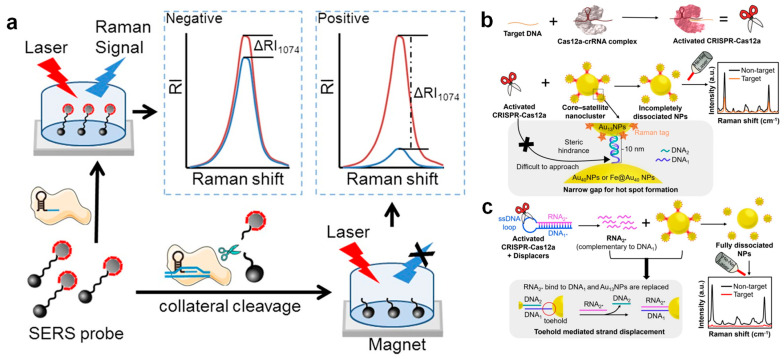
CRISPR-Cas12a–integrated SERS as a representative signal-transduction architecture. (**a**) Amplification-free SERS-based CRISPR/Cas12a platform: magnetic-bead-tethered SERS probes are collaterally cleaved by target-activated Cas12a, producing a target-dependent change in the Raman intensity at 1074 cm^−1^ between negative and positive samples. In panel (**a**), the red curve represents the Raman intensity of the negative quality-control test, whereas the blue curve represents the Raman intensity of the sample test. Target-activated Cas12a cleaves the reporter-linked probe, removes Raman tags from the magnetic beads, and decreases the Raman intensity of the sample test. ΔRI is defined as the difference between the Raman intensity of the negative control test and that of the sample test at 1074 cm^−1^. (**b**) Core–satellite Au_40_@Au_13_ nanocluster design in which an intact assembly creates a narrow gap for hot-spot formation; activated CRISPR-Cas12a only partially dissociates the cluster, producing a moderate target SERS response distinguishable from non-target. (**c**) Chimeric DNA/RNA hairpin guide variant that, upon Cas12a activation, releases satellite Au_13_ nanoparticles through toehold-mediated strand displacement, fully dissociating the cluster and switching the SERS signal between non-target and target states. Panel (**a**) adapted from Ref. [24] under CC BY 4.0. Panels (**b**,**c**) adapted from Ref. [75] under CC BY 4.0.

CRISPR-associated protein 12a (Cas12a)-integrated SERS provides a programmable form of signal transduction based on clustered regularly interspaced short palindromic repeats (CRISPR) recognition. Figure 5 illustrates this principle using representative CRISPR-Cas12a-integrated SERS platforms for nucleic acid detection [24,75]. In this design, the target nucleic acid activates Cas12a. Activated Cas12a then cleaves reporter-linked nucleic acid probes, changing the distribution or intensity of Raman reporters after magnetic separation [25,26,76]. The SERS signal therefore reports a target-triggered biochemical reaction rather than the intrinsic Raman spectrum of the target nucleic acid itself.

Figure 5 highlights this principle. CRISPR provides sequence-specific recognition, while the Raman reporter provides a strong optical output. The two functions are separated but coupled through a programmable reaction. This is particularly valuable for low-copy nucleic acid targets, where direct SERS is difficult because the target is scarce, weakly scattering, and embedded in a complex sample matrix. By translating molecular recognition into reporter release, cleavage, or accumulation, CRISPR-SERS creates a more measurable signal pathway.

Nanozyme-based SERS follows the same architectural logic through catalytic chemistry. Instead of using a reporter that is simply present or absent, nanozyme systems convert target recognition or target-induced chemical reactions into reaction-mediated signal generation. A catalytic nanoparticle or nanozyme-like substrate can transform a Raman-silent or weakly Raman-active precursor into a stronger Raman-active product, such as oxTMB, which can then be monitored by SERS [27,28,29]. In other designs, target binding or target-induced redox reactions may inhibit, release, or modulate catalytic activity. For example, Ag nanozyme-decorated metal–polyphenol nanospheres can catalyze TMB oxidation and use the redox response of glutathione to regulate the oxTMB SERS signal, allowing for catalytic SERS detection in cellular environments [28]. The result is signal amplification through turnover: one recognition event can influence many reporter molecules.

Catalytic amplification is attractive for low-abundance detection because it adds gain beyond plasmonic enhancement. But it also introduces kinetic variables. Reaction time, substrate diffusion, enzyme-like activity, pH, temperature, and local concentration all affect the final spectrum. Nanozyme-SERS is therefore not only a nanomaterial problem. It is a reaction-engineering problem. The analytical response depends on how recognition, catalytic conversion, transport, and Raman readout are coordinated in time and space.

Lateral-flow immunoassay (LFIA)-based SERS and other rapid-format platforms bring signal transduction into deployable assay formats. In these systems, SERS nanotags migrate through a strip or porous membrane, bind at a test line, and are read by a portable or benchtop Raman system [30,31,32,77]. The target is detected through accumulation of Raman reporters rather than through its own spectral fingerprint. This approach preserves the operational simplicity of lateral-flow testing while improving sensitivity and multiplexing compared with colorimetric readout.

The value of SERS-LFIA is not only lower detection limit. It shows that signal transduction must be packaged into a usable workflow. Capillary transport, test-line geometry, reporter stability, antibody density, membrane properties, and Raman acquisition position all influence the result. A strip with excellent chemistry can still perform poorly if transport is inconsistent or if the Raman reader samples the wrong region. For point-of-care SERS, the assay format is part of the sensing architecture.

Despite their different chemistries, these formats follow the same logic: they convert a biological recognition event into a spectral output that is easier to measure than the target itself. The output may be a Raman reporter peak, a barcode pattern, a cleaved probe, a catalytic product, or a test-line map. This structured output is especially important for AI-assisted SERS. Machine learning models perform better when the input data are reproducible, interpretable, and connected to a known signal pathway. Transduction-based SERS can provide such inputs more reliably than uncontrolled label-free spectra.

However, transduction does not eliminate variability. It moves the variability to different parts of the system. Nanotags can vary in reporter loading or aggregation state. Immuno-SERS can vary in antibody orientation and sandwich spacing. CRISPR-SERS depends on reaction efficiency and probe cleavage. Nanozyme-SERS depends on catalytic kinetics. LFIA-SERS depends on flow, accumulation, and reader geometry. These factors must be controlled before computational analysis can be trusted.

For this reason, signal transduction should be understood as the second major architectural step in SERS biosensing. Direct SERS asks the target to generate the spectrum. Recognition-enabled SERS asks the interface to place the target. Transduction-based SERS asks the target to trigger a designed spectral response. This progression makes the signal stronger and more structured, but it also makes the sensing system more complex.

Table 1 summarizes representative SERS biosensing platforms and their reported analytical performance. The table is intended to provide a concise comparison of sensing strategies, signal-generation formats, and performance metrics across label-free, recognition-enabled, transduction-based, digital, and AI-assisted SERS systems. 

That complexity leads naturally to the next stage. Once SERS signals are generated through structured assays, reporter patterns, digital maps, or multiplexed readouts, the remaining challenge is how to represent and interpret them reliably. Digital SERS addresses the measurement side of this problem by converting fluctuating intensity into countable events. AI/ML-assisted analysis addresses the interpretation side by extracting diagnostic meaning from complex spectral data. Together, they form the next layer of SERS biosensing architecture.

## 5. Digital SERS and AI/ML-Assisted Interpretation: From Spectral Measurement to Diagnostic Inference

Signal-transduction strategies make SERS outputs stronger and more structured. They do not, by themselves, solve the problem of measurement variability or diagnostic interpretation. A Raman reporter can fluctuate in intensity. A test line can be spatially heterogeneous. A multiplex spectrum can contain overlapping peaks. A label-free biofluid spectrum can reflect many weak biochemical changes at once. As SERS biosensing moves toward real samples and clinical decisions, the central question shifts again: how should the measured signal be represented, and how should it be interpreted?

Digital SERS and AI/ML approach this problem from different sides. Digital formats regularize the measurement itself, whereas AI/ML methods deal with the complexity of the resulting spectra. Digital SERS changes the measurement format by moving away from purely analog intensity averaging and toward countable, partitioned, or event-based readouts. AI/ML changes the interpretation format by treating spectra as high-dimensional patterns rather than as a small number of manually selected Raman bands.

In conventional SERS, quantification often depends on peak intensity. This is fragile because SERS intensity is strongly affected by hotspot heterogeneity, local reporter concentration, molecular orientation, laser focusing, and substrate variation. A small number of high-enhancement sites can dominate the measured signal. Two measurements with the same analyte concentration can therefore produce different intensities if the local hotspot distribution differs. As a result, SERS has often struggled to become a routine quantitative technique despite its high sensitivity.

Digital SERS tries to reduce this dependence on fluctuating intensity. Instead of asking how strong the signal is on average, digital approaches ask how many measurement units are positive. These units may be nanopillars, droplets, microchambers, pixels, mapped regions, or single-particle events [33,34,35,36,78]. The output becomes an event count, occupancy fraction, binary map, or probability estimate. Although this approach does not remove every source of error, it changes the statistics of the measurement. Quantification becomes less dependent on a few uncontrolled hotspots and more dependent on the number and distribution of detectable events.

**Figure 6:**
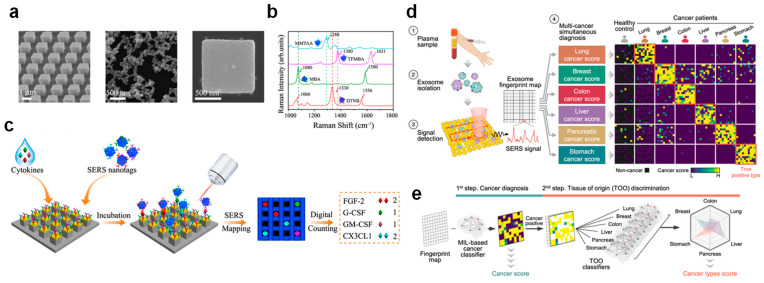
Digital SERS and AI-driven diagnostic interpretation. (**a**) SEM characterization of a patterned nanopillar SERS substrate and the SERS nanotags used for single-molecule cytokine detection. (**b**) The different-colored solid lines represent the SERS spectra of different Raman nanotags, and the vertical dashed lines indicate their characteristic Raman bands used for nanotag identification and channel assignment. (**c**) Digital single-molecule nanopillar SERS workflow: cytokines captured on the patterned substrate are tagged, SERS-mapped, and digitally counted to quantify multiplexed cytokine panels (e.g., FGF-2, G-CSF, GM-CSF, CX3CL1) for monitoring immune toxicities. (**d**) Single-test Exosome-SERS-AI pipeline: plasma exosomes are isolated, interrogated to obtain a fingerprint map, and converted into cancer scores across multiple cancer types versus healthy control. (**e**) Multiple-instance-learning-based two-step classifier that first detects cancer positivity from the fingerprint map and then discriminates tissue-of-origin (TOO) among lung, breast, colon, liver, pancreas and stomach. Panels (**a**–**c**) adapted from Ref. [36] under CC BY 4.0. Panels (**d**,**e**) adapted from Ref. [41] under CC BY 4.0.

The digital nanopillar SERS platform shown in Figure 6 illustrates this logic [36]. A large number of spatially separated plasmonic nanopillars act as discrete sensing sites. Rather than relying only on ensemble-averaged intensity, the platform detects and counts molecular events across many nanostructured units. This makes the measurement more compatible with statistical interpretation and reduces the influence of local hotspot variability. Digital SERS should not be conflated with AI. Its main contribution is to produce structured and countable measurement outputs that are easier to quantify, compare, and validate.

AI/ML-assisted SERS addresses a different limitation. Even when measurement is stable, the spectrum itself may be difficult to interpret. In biological samples, diagnostically relevant information is rarely confined to one dominant Raman band. It may be distributed across many small changes in peak position, relative intensity, baseline shape, and correlated spectral regions [38,39,40,41,42,79]. This is especially true for serum, plasma, urine, extracellular vesicles, and cells, where SERS spectra often represent a biochemical state rather than a single purified analyte.

Conventional peak-based analysis can be useful when the target peak is known, isolated, and reproducible. But many SERS biosensing problems do not satisfy those conditions. Disease-related spectral differences may be subtle. Matrix effects may shift or obscure bands. Multiplex reporters may overlap. Patient samples may vary for reasons unrelated to disease. Under these conditions, AI/ML methods become useful because they can learn patterns across the full spectral profile, provided that the data are collected and validated correctly.

The Exosome-SERS-AI example in Figure 6 represents this second direction [41]. Exosomes carry complex molecular information related to their cell of origin and disease state. Their SERS spectra are not simple single-analyte fingerprints. They are composite spectral profiles shaped by membrane components, proteins, lipids, and other vesicle-associated molecules. Deep-learning-based analysis can convert these profiles into patient-level decision-making outputs, including cancer classification and tissue-of-origin inference. This example shows why AI/ML is particularly important for label-free or minimally labeled SERS systems: the diagnostic signal is distributed across the spectrum, not concentrated in one obvious peak.

This distinction also helps separate the role of AI/ML in intrinsic and extrinsic SERS [13,44,74]. In intrinsic or label-free SERS, the measured spectrum is produced directly by the analyte and its surrounding matrix. The diagnostic information is often distributed across weak, overlapping, and sample-dependent spectral features. In this situation, AI/ML can be central to the analytical workflow because the relevant spectral pattern may not be captured by a small number of predefined peaks. By contrast, in extrinsic SERS, including many immuno-SERS and nanotag-based assays, the measured spectrum is intentionally generated by an exogenous reporter. When the reporter signal is simple and well separated, AI/ML may not add much beyond conventional calibration. Its added value appears mainly when the reporter system becomes multiplexed, when spectral cross-talk occurs, when spatial maps or strip images must be interpreted, or when assay variability must be corrected across batches and instruments.

Therefore, AI/ML should not be presented as a universal requirement for all SERS biosensors [21,44,74]. Its necessity depends on where uncertainty enters the measurement. For label-free SERS, uncertainty often comes from the biological spectrum itself. For immuno-SERS, uncertainty more often comes from reporter overlap, assay geometry, batch-to-batch variation, and deployment conditions. This difference is important because it prevents overgeneralizing AI/ML as a replacement for well-designed reporter chemistry. Instead, AI/ML should be used where it solves a real analytical problem that cannot be handled reliably by univariate or conventional multivariate analysis.

Bacterial pathogen detection is another important example in which AI/ML can substantially improve SERS or Raman spectral interpretation. Whole-cell bacterial spectra are intrinsically complex because they contain contributions from cell-wall components, proteins, lipids, nucleic acids, metabolites, and growth-state-dependent biochemical variation. In addition, bacterial spectra can vary with culture conditions, sample preparation, strain identity, antibiotic exposure, and substrate interaction. Therefore, characteristic Raman peak assignment alone is often insufficient for reliable species- or strain-level identification. Deep-learning-based Raman or SERS analysis has been used to classify pathogenic bacteria, antibiotic-treatment groups, and resistant strains from high-dimensional spectral datasets [80,81,82,83,84]. These studies show that AI/ML is particularly useful when the diagnostic information is distributed across whole-cell spectral profiles rather than concentrated in one or two isolated Raman bands. The bacterial detection literature therefore provides a useful extension of AI/ML-assisted SERS biosensing beyond cancer biomarkers and extracellular vesicles, and highlights the importance of dataset scale, biological replicates, and batch-separated validation. More recently, AI-assisted SERS has also been extended toward antibacterial theranostic systems. For example, Yuan et al. integrated AI-assisted SERS biosensing with photoactivated antibacterial therapy in an Au@Cu_2−x_Se platform for multidrug-resistant bacteria, showing that AI-SERS can be used not only for bacterial identification but also for treatment-oriented antibacterial applications [85].

In AI-assisted SERS, model development begins with the quality of the spectral dataset rather than with the choice of algorithm. Spectral acquisition must be standardized in laser wavelength, power, integration time, mapping area, and sampling density [55,57,86,87]. Substrate batches should be characterized. Patient and sample metadata must be tracked. Without these controls, the model may learn experimental artifacts rather than biological differences. In SERS, this risk is especially high because substrate batch, acquisition day, operator, preprocessing method, and sample handling can all leave strong signatures in the spectra.

Preprocessing is therefore not a minor technical step. It defines what the model will see. Baseline correction, cosmic-ray removal, smoothing, normalization, spectral range selection, and wavenumber calibration all shape the input data [57,86,88]. These procedures can improve comparability, but they can also introduce bias if chosen after inspecting class separation. For clinical AI-SERS, preprocessing should be treated as part of the analytical protocol and fixed before final validation. A model is only as trustworthy as the spectral representation used to train it.

Model selection should be guided by dataset size, spectral complexity, and the intended use of the output rather than by algorithmic novelty alone. Classical methods such as principal component analysis–linear discriminant analysis (PCA-LDA), support vector machines (SVMs), and random forests remain useful when datasets are modest and interpretability is important [40,89,90]. They can reveal whether broad spectral separation exists and which regions contribute most to classification. Deep learning methods, including one-dimensional convolutional neural networks (1D-CNNs) and related architectures, become more attractive when larger datasets are available and when diagnostic information is distributed across complex spectral patterns [39,41,79,91,92]. Transfer learning and domain adaptation are important when models must move across instruments, substrate batches, or clinical sites [72,73].

Beyond conventional classification models, several emerging AI strategies are becoming relevant to SERS biosensing. Transfer learning and domain adaptation are useful when spectra must be compared across instruments, substrate batches, or clinical sites. Self-supervised or representation-learning approaches may help leverage large unlabeled spectral datasets before limited labeled clinical data become available. Uncertainty-aware models can also be useful for diagnostic SERS because they can flag spectra that fall outside the validated training domain. Active learning and Bayesian optimization may further reduce experimental burden by guiding substrate design, spectral acquisition, or assay optimization toward the most informative conditions. These approaches are not replacements for experimental validation, but they can help make AI/ML-assisted SERS more transferable and less dependent on a single dataset, instrument, or laboratory.

Explainability is essential for AI-assisted SERS. A model with high apparent accuracy but no connection to plausible spectral or biochemical features remains difficult to interpret and difficult to validate clinically. Feature attribution methods, including SHapley Additive exPlanations (SHAP), gradient-weighted class activation mapping (Grad-CAM), and band-importance mapping, can help identify which spectral regions influence classification [44,93]. These outputs should not be treated as proof of biological mechanism by themselves, but they can help test whether a model is using plausible Raman features or hidden confounders. For a diagnostic technology, this distinction matters.

Validation is even more important. Many SERS datasets contain multiple spectra from the same sample. If spectra are randomly split into training and test sets, information from the same patient or specimen can leak across both sets. The resulting performance may look excellent but fail on new patients. For AI-assisted SERS, validation should therefore be performed at the patient or sample level. Leave-one-patient-out cross-validation (LOPO-CV), external test cohorts, independent instrument validation, and batch-separated evaluation are far more meaningful than random spectral splitting [94].

A further important issue in AI/ML-assisted SERS biosensing is the amount and structure of data required for model development. To date, many SERS studies have reported high classification accuracy by using large numbers of spectra. However, the number of spectra alone does not always represent the real size or diversity of the dataset. This is because many spectra can be collected from the same patient, the same biological sample, the same bacterial isolate, the same substrate batch, or the same acquisition session. Therefore, for AI/ML-assisted SERS, it is necessary to distinguish the number of independent samples from the number of spectral measurements. The former reflects biological, clinical, or experimental diversity, whereas the latter mainly reflects the density of spectral sampling.

This distinction is particularly important in SERS because spectral variation can originate from both the target and the measurement system. Substrate morphology, hotspot distribution, laser focusing, sample preparation, matrix composition, and preprocessing can all affect the measured spectra. If these factors are not properly separated between training and test datasets, an AI/ML model may learn substrate- or batch-specific features rather than target-related spectral information. Therefore, dataset design in AI/ML-assisted SERS should consider not only how many spectra are collected, but also how many independent samples, patients, isolates, substrate batches, instruments, and acquisition days are included.

Table 2 summarizes representative AI/ML-assisted SERS studies in which dataset size was reported. The table separates independent units, such as patients, specimens, isolates, or experimental samples, from the total number of spectra used for model development and evaluation. It also includes model type, augmentation or transfer-learning strategy, and reported performance. The surveyed studies show that the required data scale depends strongly on the analytical task. Classical chemometric approaches, such as PCA-LDA or OPLS-DA, have been applied to relatively small patient or biofluid datasets. In contrast, deep-learning models for bacterial identification, multi-cancer classification, or spectral-map analysis usually require larger spectral libraries or additional strategies such as augmentation and transfer learning.

Several points can be drawn from this comparison. First, small clinical datasets can still be useful when validation is performed at the patient or sample level, but repeated spectra from the same specimen should not be treated as independent biological samples. Second, deep-learning models can benefit from large spectral datasets, but large spectrum numbers do not remove the need for independent validation. Third, augmentation and transfer learning can reduce the experimental burden, but they should be regarded as supporting strategies rather than substitutes for real test samples. Therefore, future AI/ML-assisted SERS studies should report learning curves, independent sample numbers, spectra per sample, substrate batch information, instrument variation, and validation strategy together with model accuracy.

In this sense, the data requirement for AI/ML-assisted SERS cannot be defined by a single universal number. Instead, it should be evaluated according to the sensing format, target complexity, model class, and intended use of the output. For simple reporter-based immuno-SERS, a large AI/ML dataset may not be necessary if the reporter peak is strong, isolated, and reproducible. For label-free biofluid profiling, bacterial identification, exosome classification, or patient-level diagnosis, larger and more diverse datasets are needed because the diagnostic information is distributed across complex spectral patterns. This difference also supports the view that AI/ML is essential in some SERS formats, but optional in others.

The relationship between digital SERS and AI/ML should be understood through this validation lens. Digital measurement can reduce the uncertainty of the raw readout. AI/ML can reduce the uncertainty of interpretation. However, neither approach can overcome poorly designed validation. A digital platform with inconsistent sample handling will still be unreliable. A deep-learning model trained on biased data will still be misleading. The strongest systems will be those in which substrate control, measurement representation, spectral preprocessing, model training, and validation are designed together.

Figure 6 captures this convergence. The digital SERS example shows how measurement can be regularized through countable events. The Exosome-SERS-AI example shows how complex spectra can be converted into patient-level decision-making outputs. Together, they illustrate the core transition of the field: SERS biosensing is no longer only about producing detectable spectra, but about producing spectra that can be measured, modeled, validated, and interpreted as clinically meaningful information.

The role of AI in SERS development also changes under this framework. AI is often presented as a classifier applied after spectra are collected. That view is too narrow. In mature AI-assisted SERS systems, model outputs can feed back into experimental design. They can identify unstable substrate batches, select informative spectral regions, guide sampling density, optimize preprocessing parameters, flag uncertain predictions, and reveal when a new sample falls outside the training distribution. In this closed-loop view, AI is not only an interpretation tool [74,95,96]. It becomes a feedback layer for controlling the sensing workflow.

The promise of AI/ML-assisted SERS therefore lies not in replacing spectroscopy, but in making spectroscopic information usable under realistic conditions. Computational interpretation is most reliable when biological information is already encoded in reproducible spectral structure. This is why AI-assisted SERS must be developed as an integrated architecture rather than as a software layer attached to unstable measurements.

The next question is whether such integrated systems can function outside controlled laboratory settings. For clinical translation, high sensitivity and high model accuracy are not enough. The platform must tolerate real biofluids, operate across batches and instruments, avoid data leakage, report uncertainty, and fit into a practical workflow. These requirements are the focus of the next section.

## 6. Clinical Translation and Validation: From Analytical Performance to Deployable Systems

The previous sections describe how SERS biosensing has evolved from direct spectral response to recognition-enabled sensing, signal transduction, digital measurement, and AI/ML-assisted interpretation. Each layer improves a specific part of the problem. Substrate engineering improves the physical measurement environment. Recognition interfaces improve target localization. Transduction strategies improve signal generation. Digital SERS improves measurement representation. AI/ML improves spectral interpretation, but clinical translation ultimately depends on whether these layers remain coordinated under realistic operating conditions.

Many SERS biosensors still face difficulty at this stage. A platform may show an impressive limit of detection in buffer, a strong Raman signal on an optimized substrate, or high classification accuracy in a small dataset [97,98,99]. Such results are necessary, but they are only early indicators of clinical potential. Real diagnostic use introduces variability in patient samples, matrix composition, sample handling, substrate batches, instrument conditions, operators, and data processing. A clinically useful SERS biosensor must preserve analytical meaning across all of these sources of variation.

**Figure 7:**
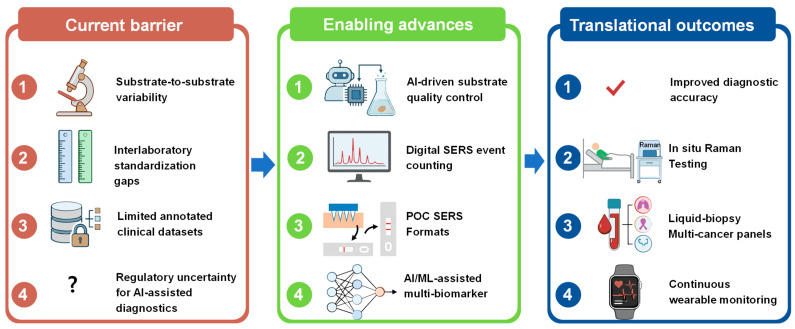
Clinical translation landscape for AI-assisted SERS biosensing. The schematic organizes the field into three stages. Current bottlenecks (**left**) include substrate-to-substrate variability, interlaboratory standardization gaps, limited annotated clinical datasets, and regulatory uncertainty for AI-assisted diagnostics. Enabling advances (**center**) span AI-driven substrate quality control, digital SERS event counting, point-of-care SERS formats, and AI/ML-assisted multi-biomarker analysis. Translational outcomes (**right**) include improved diagnostic accuracy, In situ Raman testing, liquid-biopsy multi-cancer panels, and continuous wearable monitoring.

Figure 7 summarizes this translational landscape. The left column highlights persistent barriers: substrate-to-substrate reproducibility, inter-laboratory standardization gaps, limited annotated clinical datasets, and regulatory or in vitro diagnostic validation challenges. These barriers explain why SERS has produced many strong proof-of-concept studies but fewer routine diagnostic products. The middle column shows enabling advances, including AI-driven substrate quality control, point-of-care formats, digital SERS counting, and deep-learning-based multi-cancer panels. The right column then translates these advances into possible clinical outcomes such as patient-level decision-making accuracy, bedside Raman testing, liquid-biopsy panels, and wearable monitoring.

A first requirement for translation is reproducibility. SERS is highly sensitive to nanoscale structure, which is both its advantage and its weakness. Small changes in hotspot geometry, nanoparticle aggregation, reporter loading, or surface coverage can produce large differences in signal intensity. For direct label-free SERS, this can change the measured fingerprint. For reporter-based assays, it can alter quantitative output. For AI-assisted systems, it can be even more problematic because the model may learn substrate-specific signatures rather than biological information. Reproducibility must therefore be evaluated at multiple levels: spot-to-spot, substrate-to-substrate, batch-to-batch, and instrument-to-instrument [55,87,97].

The second requirement is matrix tolerance. A SERS platform validated only in buffer is not yet a biosensor for clinical use. Serum, plasma, saliva, urine, and interstitial fluid contain abundant molecules that can foul surfaces, compete with targets, change nanoparticle behavior, and introduce spectral background. Matrix effects are especially important for label-free SERS because the analyte and background species directly compete for the same active surface [1,100,101,102]. They are also important for transduction-based systems, where nonspecific binding, altered reaction kinetics, or impaired capillary flow can distort the measured response. A translational SERS study should therefore include realistic biofluid testing as early as possible, not only as a final demonstration.

The third requirement is patient-level validation. This requirement is especially relevant for AI-assisted SERS. Many spectra can be collected from one patient, one sample, or one substrate. If these spectra are randomly divided into training and test sets, the model can indirectly learn patient-specific, batch-specific, or preparation-specific features that appear in both sets. The reported accuracy may then be artificially high. For clinical AI-SERS, validation should be performed at the sample or patient level. LOPO-CV, independent test cohorts, multi-site datasets, and batch-separated validation provide stronger evidence than random spectral splitting [41,89,94].

The fourth requirement is standardization of the measurement workflow. SERS data are shaped by laser wavelength, laser power, integration time, objective lens, mapping strategy, spectral range, preprocessing method, substrate storage, and environmental conditions. Without harmonization, spectra collected on different days or instruments may not be directly comparable [87,95]. The issue is both hardware-related and computational. Baseline correction, normalization, smoothing, and spectral alignment can change model input substantially. For AI-assisted SERS, preprocessing should therefore be part of the locked analytical protocol, not a flexible step adjusted after model performance is inspected.

Calibration transfer is closely related to this issue. A model trained on one Raman instrument or substrate batch may fail when applied to another. This is one of the major obstacles to real-world deployment. Transfer learning, domain adaptation, internal standards, and cross-instrument calibration methods can help, but they must be validated experimentally [72,73,103]. The goal is not to make every instrument identical. The goal is to make the analytical output stable enough that a diagnostic decision does not depend on the particular device, batch, or acquisition site.

Usability is another translational constraint. A SERS biosensor may be analytically strong but impractical if it requires complicated sample preparation, large sample volume, long assay time, unstable reagents, or expert operation. This is why LFIA-SERS, paper-based devices, microfluidic systems, microneedle patches, and handheld Raman readers are important [30,31,32,104,105,106,107]. They reduce the distance between laboratory measurement and real use. However, simplified formats also introduce new variables, including flow uniformity, reader alignment, tissue contact, evaporation, and environmental conditions. Translation therefore requires balancing analytical sophistication with workflow simplicity.

Regulatory readiness is the final layer. For conventional SERS assays, regulatory evaluation already requires evidence of analytical sensitivity, specificity, reproducibility, stability, and clinical relevance. AI-assisted SERS adds further requirements. The diagnostic output depends on both the measurement system and the model. This means that dataset composition, training strategy, performance reporting, explainability, software updates, model drift, and post-deployment monitoring become part of the diagnostic system [108,109]. Reporting guidelines such as Standards for Reporting Diagnostic Accuracy Studies–Artificial Intelligence (STARD-AI) are therefore relevant to AI-SERS studies, especially when AI/ML-assisted SERS is evaluated as a diagnostic accuracy system [94]. Even when the sensing chemistry itself is the main novelty, dataset definition, preprocessing, test-set composition, model evaluation, bias, applicability, and generalizability should be reported clearly.

Explainability also matters at this stage. A clinically deployed AI-SERS model should not only output a class label. It should provide enough information to support trust and error analysis. Feature-attribution maps, important Raman bands, uncertainty scores, and comparison with gold-standard diagnostics can help determine whether the model is using meaningful spectral information or hidden confounders. Explainability does not make a model automatically correct, but it gives researchers and clinicians a way to interrogate model behavior.

Figure 7 emphasizes a point that is often underestimated in SERS development: translation should be treated as a design constraint, not as a final demonstration added after sensor optimization. If a substrate cannot be reproduced, it will be difficult to validate. If the interface fails in real biofluids, AI cannot correct it reliably. If the dataset is small or biased, high accuracy will not generalize. If the workflow is too complex, point-of-care use will remain unrealistic. Translation therefore requires coordinated progress in materials, interfaces, assay design, measurement protocols, AI validation, and regulatory documentation.

In this sense, the most important future SERS biosensors may not be those with the lowest reported LOD. They may be the ones that remain stable across samples, batches, instruments, and users. They may produce slightly less spectacular analytical numbers but more reliable diagnostic information. This is particularly true for AI-assisted systems, where reproducibility and validation determine whether model outputs can be trusted.

SERS biosensing is therefore moving toward a new evaluation standard. Sensitivity remains important, but it is not sufficient. A mature system must show matrix tolerance, reproducibility, patient-level validation, calibration transfer, usability, and interpretable decision-making. These are not separate afterthoughts. They are the conditions under which SERS can move from a powerful spectroscopic method to a deployable diagnostic platform.

## 7. Future Perspectives: Toward AI-Assisted SERS as a Diagnostic System

The future of SERS biosensing will not be determined by enhancement factor alone. Stronger hotspots will continue to matter, but they will not be sufficient. The next stage of the field will depend on whether SERS platforms can generate spectra that are reproducible, interpretable, transferable, and clinically meaningful. Therefore, future AI-assisted SERS systems should be evaluated not only by model performance, but also by the function of the model, the main source of risk, and the validation strategy, as summarized in Table 3.

One area where AI/ML can have a practical impact is the optimization of substrates and interfaces. Most SERS substrates are still developed through empirical trial-and-error [11,12,45]. Nanoparticle size, gap distance, surface chemistry, probe density, linker length, antifouling coating, and assay geometry are varied experimentally until an acceptable response is obtained. This approach can work, but it is inefficient and often difficult to generalize. AI-guided optimization can help identify design regions where enhancement, uniformity, target access, and matrix resistance are balanced rather than optimized separately [74,95,96].

The best SERS substrate is not always the one with the highest signal. For clinical biosensing, a moderately enhanced but highly reproducible substrate may be more useful than an extremely strong but heterogeneous one. AI-guided design can support this shift by optimizing multiple objectives at once: intensity, reproducibility, fabrication tolerance, surface stability, and compatibility with downstream analysis. In this sense, AI becomes useful before spectral classification. It can help decide what kind of SERS data should be generated in the first place.

Another unresolved issue is whether AI-SERS models can remain valid when measurements are collected on different instruments, substrate batches, or clinical sites. Current models often perform well under the conditions in which they are trained, but real deployment requires transfer across instruments, substrate batches, acquisition days, operators, and clinical sites. This is a difficult problem because SERS spectra can carry hidden signatures of experimental conditions. A model may learn the difference between batches rather than the difference between disease states. Future systems will therefore need calibration transfer, domain adaptation, uncertainty estimation, and external validation as standard components of the workflow [72,73].

Clinical use depends strongly on this capability. A diagnostic model should not require complete retraining every time a new Raman instrument, substrate batch, or sample matrix is introduced. Instead, it should either remain stable under these changes or report when the new data fall outside the validated domain. This will require better metadata, standardized acquisition protocols, internal standards, and model-monitoring strategies. Without these elements, AI-assisted SERS may remain impressive in individual studies but fragile in real-world deployment.

The field also needs a more deliberate view of the data structures generated by SERS platforms. AI does not simply need more spectra; it needs data structures that are reproducible, traceable, and compatible with validation. Different SERS architectures naturally produce different data formats: label-free SERS provides full-spectrum biochemical profiles, reporter-based assays generate defined peaks or barcode patterns, digital SERS produces countable events, lateral-flow and microfluidic formats generate spatially resolved spectral maps, and wearable or in situ platforms can provide time-series measurements. These outputs differ in dimensionality, reproducibility, and suitability for AI/ML-assisted interpretation [33,35,36,78,106]. The design goal is therefore not only stronger signal generation, but structured data generation for reliable downstream analysis.

Digital SERS is particularly important in this context. By converting fluctuating analog intensity into countable events, digital formats provide a measurement structure that is naturally compatible with statistical modeling. Microfluidic and droplet-based SERS can add control over reaction time, sample volume, and compartmentalization. Wearable and minimally invasive formats can extend SERS from single-time-point assays toward continuous or repeated monitoring [106]. Multimodal platforms can combine Raman signals with electrochemical, optical, or immunological readouts, giving AI models more than one type of evidence to interpret [22].

A further priority is explainable and regulatory-ready AI-SERS. High classification accuracy is not enough for clinical adoption. A model should be able to show which spectral regions influenced its decision, whether those regions are chemically plausible, and how confident the model is in the output. Explainable AI does not replace biological validation, but it can help identify whether the model is learning meaningful spectral signatures or hidden confounders. Its importance will increase as SERS models move from retrospective datasets to prospective clinical studies.

Regulatory readiness should also be treated as an engineering requirement rather than a final documentation step. AI-assisted SERS systems combine hardware, chemistry, optics, software, and clinical interpretation. Changes in any one of these layers can affect the diagnostic output. Future platforms will therefore need locked preprocessing pipelines, predefined validation strategies, transparent reporting, model update plans, and post-deployment monitoring. Clinical AI reporting guidelines such as STARD-AI provide useful guidance, but the SERS community will need to translate these principles into spectroscopy-specific practice [94]. In particular, future AI-SERS studies should describe how spectra are collected, preprocessed, partitioned, validated, and interpreted across samples, instruments, and clinical settings.

The most promising future systems will likely be closed-loop architectures. In such systems, AI will not only classify spectra after acquisition. It will also guide substrate quality control, select sampling regions, monitor spectral drift, recommend recalibration, flag uncertain predictions, and inform assay redesign. This feedback loop could make SERS biosensing more robust than a one-way workflow in which materials are fabricated, spectra are collected, and models are trained independently.

The long-term goal is not simply to make SERS more sensitive. It is to make SERS more dependable. A clinically useful platform must preserve analytical meaning across real samples, devices, users, and time. This is the point at which AI-assisted SERS can move beyond being a high-performance laboratory method and become a practical diagnostic technology.

## 8. Conclusions

SERS biosensing has often been discussed through the lens of enhancement. In complex biological fluids, that view is incomplete. A useful SERS biosensor must not only generate a detectable Raman signal, but also control where the target is located, how the response is produced, how variability is suppressed, and how the final spectrum is interpreted.

Direct label-free SERS provides the most chemically transparent readout, but it also exposes the instability of hotspot-dependent sensing. Bio-recognition interfaces improve target localization, and transduction-based assays convert recognition events into more stable spectral outputs. Digital SERS further regularizes measurement by shifting part of the readout from analog intensity to countable events. AI/ML-assisted interpretation can then add an important analytical layer for full-spectrum classification, calibration transfer, explainability, and patient-level decision-making.

The main implication is that AI alone does not make SERS clinically useful. AI-assisted SERS will be reliable only when the underlying measurement is reproducible, biologically traceable, and validated at the proper sample or patient level. For this reason, substrate design, interface chemistry, assay format, spectral acquisition, preprocessing, and model validation should be developed as parts of one system.

Future progress will likely depend less on isolated records in sensitivity and more on workflows that remain stable across real samples, batches, instruments, and clinical sites. With this level of control, AI-assisted SERS may move from impressive spectroscopic demonstrations toward practical tools for liquid biopsy, infectious disease testing, point-of-care diagnostics, and longitudinal monitoring.

## Figures and Tables

**Table 1:** Representative SERS biosensing platforms and reported analytical performance.

Target/Sample	SERS Strategy	Signal or Analysis Type	Reported Performance	Ref.
Urine metabolites/urine	Label-free SERS	Spectral profiling	Cancer discrimination	[54]
Single protein	Gap-mode SERS	Direct spectral response	Single-protein-level detection	[51]
α-Fetoprotein	Immuno-SERS	Au–Ag nanostar probe	High-sensitivity biomarker detection	[14]
Thyroglobulin	Sandwich immuno-SERS	Raman reporter immunoassay	7 pg/mL	[71]
Gastric cancer exosomes	Aptamer-SERS + bHCR	Amplified aptamer scaffold	Single-exosome-level sensitivity	[67]
Enrofloxacin	MIP-SERS	Imprinted capture + SERS	Selective detection	[68]
SARS-CoV-2 RNA	CRISPR/Cas12a-SERS	Reporter cleavage/release	1 fM	[24]
Salmonella typhimurium	Nanozyme-SERS	Catalytic amplification	10 CFU/mL	[27]
PSA/CA19-9	SERS-LFIA	Test-line nanotag accumulation	8.0 × 10^−3^ ng/mL (PSA); 5.4 × 10^−2^ U/mL (CA19-9)	[31]
Cytokines	Digital SERS	Nanopillar event counting	Attomolar-range cytokine detection	[36]
Plasma exosomes/multi-cancer	Exosome-SERS-AI	Deep-learning classification	Patient-level cancer classification	[41]

Abbreviations: bHCR, branched hybridization chain reaction; PSA, prostate-specific antigen; CA19-9, carbohydrate antigen 19-9; POC, point-of-care; PCA-LDA, principal component analysis–linear discriminant analysis; AUC, area under the receiver operating characteristic curve.

**Table 2:** Reported dataset sizes in representative AI/ML-assisted SERS studies. Samples denote independent biological subjects, isolates, specimens, or experimental units, whereas spectra denote the total spectral measurements used for model development and evaluation. The table highlights that dataset requirements depend on the sensing task, model class, sample heterogeneity, augmentation or transfer-learning strategy, and validation design.

Application Domain	Samples (Independent Units)	Spectra (Training + Evaluation)	Model	Augmentation/Transfer Learning	Reported Performance	Ref.
Bacteria—species and antibiotic identification	30 isolates	60,000 (2000/isolate) + 100 fine-tune + 100 test	1D-ResNet (CNN)	Transfer learning across batches	82.2% isolate-level; 97.0% antibiotic-group accuracy	[80]
Bacteria—clinical blood pathogens	8 species (clinical isolates)	11,774	Vision Transformer (ViT) + transfer learning	Yes—Gram-positive model reused for MRSA/MSSA with only 200 spectra (98.5%)	Gram type 99.30%; species 97.56%	[81]
Bacteria—metabolite-level interpretable	8 species, 10 independent days	8000 (1000/species; 100 spectra × 10 days)	CNN + Random Forest + SVM	No; day-stratified design	Accuracy > 90%; AUC > 0.99	[82]
Multi-cancer diagnosis—plasma exosomes	753 patients (train 233/test 520)	23,051 (HC 4943 + cancer 18,108)	1D-CNN binary + tissue-of-origin	None; empirical saturation at 30–40 samples/class	Sensitivity 90.2% at specificity 94.4%; TOO mean AUC 0.945	[41]
Serum cancer (4 groups)	110 patients (30 HC + 30 BC + 30 AC + 20 AML)	110 → 1100 after augmentation	1D-CNN	Yes—random linear combination	98.27% accuracy	[42]
Head and neck cancer—cerumen	13 donors (6 CTRL + 7 HNC)	~1238 (~100/donor)	PCA-LDA (classical ML)	None	87% balanced accuracy; AUC 0.90	[89]
Pancreatic and prostate cancer—urine	74 subjects (30 NC + 22 PC + 22 PrC)	Multiple spectra/subject	PCA + OPLS-DA (classical ML)	None	100% sensitivity/100% specificity across PC, PrC, NC	[54]
COVID-19—saliva and nasopharyngeal swab	289 samples (175 saliva + 114 swab)	289 spectra; 3062 spectral features per spectrum	Random Forest; PCA/UMAP + Gaussian process	Dimensionality reduction (PCA 11/UMAP 4)	RF precision 94.1%; recall 88.9%	[83]
COVID-19—SARS-CoV-2 proteins (DeepATsers)	5 protein groups	126 → 780 after GAN augmentation	1D-CNN	GAN-based augmentation	Accuracy 60% → 97.5% after augmentation	[84]
Substrate quality control	N/A; substrate spectra	1995 (940 good + 936 bad + 119 misc.)	XGBoost (classical ML)	None	Successful good/bad spectrum filtering	[56]
Single-molecule quantification	Concentration sweep	1–100 8 × 8-pixel maps per concentration × 32 × augmentation	CNN with transfer learning	Yes—extensive augmentation	Quantification in single-molecule regime	[37]

**Table 3:** Functional roles of AI/ML in SERS biosensing and corresponding validation requirements.

AI/ML Role	Typical Methods	Main Risk	Recommended Validation
Spectral preprocessing	Baseline correction, smoothing, normalization	Preprocessing choices may introduce bias	Lock preprocessing before final testing
Feature extraction	PCA, band selection, feature attribution	Selected features may reflect batch effects	Test feature stability across batches
Classical ML classification	PCA-LDA, SVM, random forest	Inflated performance from spectral-level splitting	Patient- or sample-level validation
Deep learning	1D-CNN, ResNet-type models	Requires larger datasets; limited interpretability	External cohort and batch-separated testing
Calibration transfer	Domain adaptation, transfer learning, instrument correction	Model may fail on unseen instruments	Independent instrument validation
Explainable AI	SHAP, Grad-CAM, band-importance mapping	Attribution may not represent mechanism	Compare with plausible Raman assignments
Uncertainty estimation	Ensemble models, calibrated probabilities	Overconfident predictions outside training domain	Report confidence and out-of-distribution behavior
Closed-loop optimization	Bayesian optimization, active learning	Optimization may follow model artifacts	Experimental confirmation of optimized conditions

## Data Availability

No new data were created or analyzed in this study.

## Abbreviations

SERS	Surface-enhanced Raman scattering
AI	Artificial intelligence
ML	Machine learning
AI/ML	Artificial intelligence/machine learning
POC	Point-of-care
LOD	Limit of detection
RSD	Relative standard deviation
EV	Extracellular vesicle
MIP	Molecularly imprinted polymer
CRISPR	Clustered regularly interspaced short palindromic repeats
Cas12a	CRISPR-associated protein 12a
LFIA	Lateral-flow immunoassay
NPoM	Nanoparticle-on-mirror
PCA	Principal component analysis
LDA	Linear discriminant analysis
PCA-LDA	Principal component analysis–linear discriminant analysis
SVM	Support vector machine
CNN	Convolutional neural network
1D-CNN	One-dimensional convolutional neural network
XAI	Explainable artificial intelligence
SHAP	SHapley Additive exPlanations
Grad-CAM	Gradient-weighted class activation mapping
LOPO-CV	Leave-one-patient-out cross-validation
ROC	Receiver operating characteristic
AUC	Area under the receiver operating characteristic curve
SaMD	Software as a medical device
STARD-AI	Standards for Reporting Diagnostic Accuracy Studies–Artificial Intelligence
bHCR	Branched hybridization chain reaction
PSA	Prostate-specific antigen
CA19-9	Carbohydrate antigen 19-9

## References

[1] Langer J., Jimenez de Aberasturi D., Aizpurua J., Alvarez-Puebla R.A., Auguié B., Baumberg J.J., Bazan G.C., Bell S.E.J., Boisen A., Brolo A.G. (2020). Present and Future of Surface-Enhanced Raman Scattering. ACS Nano.

[2] Cialla-May D., Bonifacio A., Bocklitz T., Markin A., Markina N., Fornasaro S., Dwivedi A., Dib T., Farnesi E., Liu C. (2024). Biomedical SERS—The current state and future trends. Chem. Soc. Rev..

[3] Jamieson L.E., Asiala S.M., Gracie K., Faulds K., Graham D. (2017). Bioanalytical Measurements Enabled by Surface-Enhanced Raman Scattering (SERS) Probes. Annu. Rev. Anal. Chem..

[4] Zheng X.S., Jahn I.J., Weber K., Cialla-May D., Popp J. (2018). Label-free SERS in biological and biomedical applications: Recent progress, current challenges and opportunities. Spectrochim. Acta A Mol. Biomol. Spectrosc..

[5] Chen M., Zhao M., Cai Y., Zhang Q., Peng Z., Li Q., Wang Z. (2026). Surface-enhanced Raman spectroscopy for label-free cancer liquid biopsy: From fundamentals to clinical analysis of biofluid. Front. Chem..

[6] Panikar S.S., Cialla-May D., De la Rosa E., Salas P., Popp J. (2021). Towards translation of surface-enhanced Raman spectroscopy (SERS) to clinical practice: Progress and trends. TrAC Trends Anal. Chem..

[7] Cialla-May D., Zheng X.-S., Weber K., Popp J. (2017). Recent progress in surface-enhanced Raman spectroscopy for biological and biomedical applications: From cells to clinics. Chem. Soc. Rev..

[8] Schlücker S. (2014). Surface-enhanced Raman spectroscopy: Concepts and chemical applications. Angew. Chem. Int. Ed..

[9] Le Ru E.C., Blackie E., Meyer M., Etchegoin P.G. (2007). Surface enhanced Raman scattering enhancement factors: A comprehensive study. J. Phys. Chem. C.

[10] Constantinou M., Hadjigeorgiou K., Abalde-Cela S., Andreou C. (2022). Label-Free Sensing with Metal Nanostructure-Based Surface-Enhanced Raman Spectroscopy for Cancer Diagnosis. ACS Appl. Nano Mater..

[11] Lee H.K., Lee Y.H., Koh C.S.L., Phan-Quang G.C., Han X., Lay C.L., Sim H.Y.F., Kao Y.-C., An Q., Ling X.Y. (2019). Designing surface-enhanced Raman scattering (SERS) platforms beyond hotspot engineering: Emerging opportunities in analyte manipulations and hybrid materials. Chem. Soc. Rev..

[12] Ding S.-Y., You E.-M., Tian Z.-Q., Moskovits M. (2017). Electromagnetic theories of surface-enhanced Raman spectroscopy. Chem. Soc. Rev..

[13] Choi N., Zhang Y., Wang Y., Schlücker S. (2024). iSERS: From nanotag design to protein assays and ex vivo imaging. Chem. Soc. Rev..

[14] García-Ramírez J.I., Luna-Cervantes M., Izaguirre-Hernández I.Y., Hernández-Torres J., Juárez-Aguilar E., Thomas-Dupont P., Remes-Troche J.M., Zamora-Peredo L. (2025). SERS-Based Immunoassay for α-Fetoprotein Biomarker Detection Using an Au-Ag Nanostars Platform. Biosensors.

[15] Muhammad M., Huang Q. (2021). A review of aptamer-based SERS biosensors: Design strategies and applications. Talanta.

[16] Pavan S., Berti F. (2012). Short peptides as biosensor transducers. Anal. Bioanal. Chem..

[17] Mosier-Boss P.A. (2017). Review of SERS Substrates for Chemical Sensing. Nanomaterials.

[18] Granger J.H., Granger M.C., Firpo M.A., Mulvihill S.J., Porter M.D. (2013). Toward development of a surface-enhanced Raman scattering (SERS)-based cancer diagnostic immunoassay panel. Analyst.

[19] Chen D., Ma Y., Yang A., Hu L., Zhou H., Xu J., Chen S., Nie D., Feng W., Cai H. (2025). Dual-Enhanced SERS Satellite Immuno-Nanocomplex for Multiple PSA-Mediated PHI Assay Toward Clinical Prostate Cancer Screening. Adv. Sci..

[20] Chen X., Liu T., Chen W., Lin Z., Zhang Q., Zhou B., Zhou X. (2026). From nanotags to precision biomedicine: SERS-driven progress and innovation in tumor biomarker profiling, dynamic bioimaging, AI-enhanced diagnostics and therapy. Theranostics.

[21] Laing S., Sloan-Dennison S., Faulds K., Graham D. (2025). Surface Enhanced Raman Scattering for Biomolecular Sensing in Human Healthcare Monitoring. ACS Nano.

[22] Hu C., Guan M., Mi F., Zhang S., Geng P. (2026). Multimodal SERS Biosensing Platforms: Emerging Opportunities for Ultrasensitive Biomarker Detection and Intelligent Diagnostics. ACS Sens..

[23] Lin D., Hsieh C.-L., Hsu K.-C., Liao P.-H., Qiu S., Gong T., Yong K.-T., Feng S., Kong K.V. (2021). Geometrically encoded SERS nanobarcodes for the logical detection of nasopharyngeal carcinoma-related progression biomarkers. Nat. Commun..

[24] Liang J., Teng P., Xiao W., He G., Song Q., Zhang Y., Peng B., Li G., Hu L., Cao D. (2021). Application of the amplification-free SERS-based CRISPR/Cas12a platform in the identification of SARS-CoV-2 from clinical samples. J. Nanobiotechnol..

[25] Wang H., Su A., Bao C., Liang C., Xu W., Chang J., Xu S. (2024). A CRISPR/Cas12a-SERS platform for amplification-free gene detection of African swine fever virus. Talanta.

[26] Rabiee N. (2026). SERS-Enhanced CRISPR Biosensors: A Platform for Ultrasensitive Molecular Diagnostics. Anal. Chem..

[27] Li Z., Hu J., Zhan Y., Shao Z., Gao M., Yao Q., Li Z., Sun S., Wang L. (2023). Coupling Bifunctional Nanozyme-Mediated Catalytic Signal Amplification and Label-Free SERS with Immunoassays for Ultrasensitive Detection of Pathogens in Milk Samples. Anal. Chem..

[28] Li Y., Li P., Chen Y., Wu Y., Wei J. (2023). Interfacial Deposition of Ag Nanozyme on Metal-Polyphenol Nanosphere for SERS Detection of Cellular Glutathione. Biosens. Bioelectron..

[29] Wang X., Sheng J., Yang H., Shen K., Yao J., Qian Y., Chen G. (2025). Au@Pt@HP1-HP2@Fe3O4 Nanoenzymatic Complexes Based on CHA Signal Amplification Strategy for Ultrasensitive SERS Detection of ctDNA in Liver Cancer. Int. J. Nanomed..

[30] Hwang J., Lee S., Choo J. (2016). Application of a SERS-based lateral flow immunoassay strip for the rapid and sensitive detection of staphylococcal enterotoxin B. Nanoscale.

[31] Park S., Jeong Y., Jang S., Yang C.-H., Chu J., Kang H., Park S., Chang H., Jun B.-H. (2026). Multiplexed Detection of Cancer Biomarker Using a Dual-Mode Colorimetric-SERS Lateral Flow Immunoassay Based on Elongated Rod Ag Nanoshell (ERNS) SERS Tags. Biosensors.

[32] Zhi W., Wang L., Dai L., Xu J., He T., Zong X., Xu J., Cai H., Pi J., Sun P. (2025). SERS-based lateral flow immunoassay for rapid and sensitive sensing of nucleocapsid protein toward SARS-CoV-2 screening in clinical samples. Anal. Chim. Acta.

[33] Bi X., Czajkowsky D.M., Shao Z., Ye J. (2024). Digital colloid-enhanced Raman spectroscopy by single-molecule counting. Nature.

[34] Ando J., Asanuma M., Dodo K., Fujita H., Yamakoshi H., Sodeoka M., Watanabe T.M. (2025). Digital SERS bioanalysis of single-enzyme biomarkers. Proc. Natl. Acad. Sci. USA.

[35] Wang W., Srivastava S., Garg A., Xiao C., Hawks S., Pan J., Duggal N., Isaacman-VanWertz G., Zhou W., Marr L.C. (2024). Digital Surface-Enhanced Raman Spectroscopy–Lateral Flow Test Dipstick: Ultrasensitive, Rapid Virus Quantification in Environmental Dust. Environ. Sci. Technol..

[36] Li J., Wuethrich A., Edwardraja S., Lobb R.J., Puttick S., Rose S., Trau M. (2021). A digital single-molecule nanopillar SERS platform for predicting and monitoring immune toxicities in immunotherapy. Nat. Commun..

[37] Thrift W.J., Ragan R. (2019). Quantification of Analyte Concentration in the Single Molecule Regime Using Convolutional Neural Networks. Anal. Chem..

[38] Dong S., He D., Zhang Q., Huang C., Hu Z., Zhang C., Nie L., Wang K., Luo W., Yu J. (2023). Early cancer detection by serum biomolecular fingerprinting spectroscopy with machine learning. eLight.

[39] Shi Y., Shi W., Sun M., Lin L., Ye J. (2023). Early cancer detection by SERS spectroscopy and machine learning. Light Sci. Appl..

[40] Srivastava S., Garg A., Xiao C., Hawks S., Pan J., Duggal N., Isaacman-VanWertz G., Zhou W., Marr L.C., Vikesland P.J. (2024). Machine Learning-Assisted Surface-Enhanced Raman Spectroscopy Detection for Environmental Applications: A Review. Environ. Sci. Technol..

[41] Shin H., Choi B.H., Shim O., Kim J., Park Y., Cho S.K., Kim H.K., Choi Y. (2023). Single test-based diagnosis of multiple cancer types using Exosome-SERS-AI for early stage cancers. Nat. Commun..

[42] Xiong C.-C., Zhu S.-S., Yan D.-H., Yao Y.-D., Zhang Z., Zhang G.-J., Chen S. (2023). Rapid and precise detection of cancers via label-free SERS and deep learning. Anal. Bioanal. Chem..

[43] Diao X., Li X., Hou S., Li H., Qi G., Jin Y. (2023). Machine Learning-Based Label-Free SERS Profiling of Exosomes for Accurate Fuzzy Diagnosis of Cancer and Dynamic Monitoring of Drug Therapeutic Processes. Anal. Chem..

[44] Quarin S.M., Vang D., Dima R.I., Stan G., Strobbia P. (2025). AI in SERS sensing moving from discriminative to generative. npj Biosens..

[45] Das G.M., Managò S., Mangini M., De Luca A.C. (2021). Biosensing Using SERS Active Gold Nanostructures. Nanomaterials.

[46] Moskovits M. (2005). Surface-enhanced Raman spectroscopy: A brief retrospective. J. Raman Spectrosc..

[47] Guerrini L., Graham D. (2012). Molecularly-mediated assemblies of plasmonic nanoparticles for surface-enhanced Raman spectroscopy applications. Chem. Soc. Rev..

[48] Tian L., Su M., Yu F., Xu Y., Li X., Li L., Liu H., Tan W. (2018). Liquid-state quantitative SERS analyzer on self-ordered metal liquid-like plasmonic arrays. Nat. Commun..

[49] Benz F., Schmidt M.K., Dreismann A., Chikkaraddy R., Zhang Y., Demetriadou A., Carnegie C., Ohadi H., de Nijs B., Esteban R. (2016). Single-molecule optomechanics in “picocavities”. Science.

[50] de Nijs B., Bowman R.W., Herrmann L.O., Benz F., Barrow S.J., Sigle D., Mertens J., Oulton R., Esteban R., Aizpurua J. (2016). SERS of Individual Nanoparticles on a Mirror: Size Does Matter, but so does Shape. J. Phys. Chem. Lett..

[51] Schuknecht F., Kołątaj K., Steinberger M., Liedl T., Lohmueller T. (2023). Accessible hotspots for single-protein SERS in DNA-origami assembled gold nanorod dimers with tip-to-tip alignment. Nat. Commun..

[52] Lin L.L., Alvarez-Puebla R., Liz-Marzán L.M., Trau M., Wang J., Fabris L., Wang X., Liu G., Xu S., Han X.X. (2025). Surface-Enhanced Raman Spectroscopy for Biomedical Applications: Recent Advances and Future Challenges. ACS Appl. Mater. Interfaces.

[53] Liu Y., Li M., Liu H., Kang C., Wang C. (2024). Cancer diagnosis using label-free SERS-based exosome analysis. Theranostics.

[54] Phyo J.B., Woo A., Yu H.J., Lim K., Cho B.H., Jung H.S., Lee M.-Y. (2021). Label-Free SERS Analysis of Urine Using a 3D-Stacked AgNW-Glass Fiber Filter Sensor for the Diagnosis of Pancreatic Cancer and Prostate Cancer. Anal. Chem..

[55] Sloan-Dennison S., Wallace G.Q., Hassanain W.A., Laing S., Faulds K., Graham D. (2024). Advancing SERS as a quantitative technique: Challenges, considerations, and correlative approaches to aid validation. Nano Converg..

[56] Rojalin T., Antonio D., Kulkarni A., Carney R.P. (2022). Machine Learning-Assisted Sampling of Surface-Enhanced Raman Scattering (SERS) Substrates Improves Data Collection Efficiency. Appl. Spectrosc..

[57] Yan C., Li Y., Zhang X., Liang M., Zhang J., Zhang Y. (2025). A review on spectral data preprocessing techniques for machine learning and quantitative analysis. iScience.

[58] Hashemi P., Afkhami A., Baradaran B., Halabian R., Madrakian T., Arduini F., Nguyen T.A., Bagheri H. (2020). Well-Orientation Strategy for Direct Immobilization of Antibodies: Development of the Immunosensor Using the Boronic Acid-Modified Magnetic Graphene Nanoribbons for Ultrasensitive Detection of Lymphoma Cancer Cells. Anal. Chem..

[59] Huang J., Xie Z., Xie L., Luo S., Zeng T., Zhang Y., Zhang M., Wang S., Li M., Wei Y. (2022). Explore how immobilization strategies affected immunosensor performance by comparing four methods for antibody immobilization on electrode surfaces. Sci. Rep..

[60] Simões B., Guedens W.J., Keene C., Kubiak-Ossowska K., Mulheran P., Kotowska A.M., Scurr D.J., Alexander M.R., Broisat A., Johnson S. (2021). Direct Immobilization of Engineered Nanobodies on Gold Sensors. ACS Appl. Mater. Interfaces.

[61] Çapkın E., Kutlu A., Yüce M. (2023). Repurposing Fc Gamma Receptor I (FcγRI, CD64) for Site-Oriented Monoclonal Antibody Capture: A Proof-of-Concept Study for Real-Time Detection of TNF-α. Heliyon.

[62] Du Q., Wang W., Zeng X., Luo X. (2023). Antifouling zwitterionic peptide hydrogel based electrochemical biosensor for reliable detection of prostate specific antigen in human serum. Anal. Chim. Acta.

[63] Stefancu A., Moisoiu V., Couti R., Andras I., Rahota R., Crisan D., Pavel I.E., Socaciu C., Leopold N., Crisan N. (2018). Combining SERS analysis of serum with PSA levels for improving the detection of prostate cancer. Nanomedicine.

[64] Garg A., Zong Z., Vikesland P., Gloag E.S., Zhou W. (2025). Zwitterionic nanoplasmonic bio-meshes for in situ spatiotemporal SERS monitoring of Pseudomonas aeruginosa biofilms. npj Biosens..

[65] Ji B., Liu Z., Lv Z., Yang Q., Sun J., Su G., Xia Y., Yan X., Hu J., Hu P. (2025). Targeted molecular rapid SERS diagnosis in clinical human serum through aptamer origami-collapsed nanofingers chip. Biosens. Bioelectron..

[66] Chen C., Wang X., Wang X., Waterhouse G.I.N., Wang R., Sun Y., Xu Z. (2024). SERS aptasensor utilizing aptamer-conformation-mediated regulation of Au dumbbell dimers for the ultrasensitive detection of β-phenethylamine in foods. Sens. Actuators B Chem..

[67] Liu X., Zhang J., Chen Z., He X., Yan C., Lv H., Chen Z., Liu Y., Wang L., Song C. (2025). Branched hybridization chain reaction and tetrahedral DNA-based trivalent aptamer powered SERS sensor for ultra-highly sensitive detection of cancer-derived exosomes. Biosens. Bioelectron..

[68] Neng J., Wang Y., Zhang Y., Chen P., Yang K. (2023). MIPs–SERS Sensor Based on Ag NPs Film for Selective Detection of Enrofloxacin in Food. Biosensors.

[69] Gu Z., Zhao D., He H., Wang Z. (2024). SERS-Based Microneedle Biosensor for In Situ and Sensitive Detection of Tyrosinase. Biosensors.

[70] Wang Y., Yan B., Chen L. (2013). SERS Tags: Novel Optical Nanoprobes for Bioanalysis. Chem. Rev..

[71] Spaziani S., Quero G., Managò S., Zito G., Terracciano D., Macchia P.E., Galeotti F., Pisco M., De Luca A.C., Cusano A. (2023). SERS assisted sandwich immunoassay platforms for ultrasensitive and selective detection of human Thyroglobulin. Biosens. Bioelectron..

[72] Wang T., Yang Y., Lu H., Cui J., Chen X., Ma P., Zhong W., Zhao Y. (2025). Functional regression for SERS spectrum transformation across diverse instruments. Analyst.

[73] Lai J., Li M., Chen S., Long J., Chen Y., Lu H., Zou C., Zhang Z. (2025). Calibration Transfer of Deep Learning Models among Multiple Raman Spectrometers via Low-Rank Adaptation. Anal. Chem..

[74] Geddis A., Williams H., Bashir S., Malenfant J., Dubois C., Hamlet L., Masson J.-F. (2026). Artificial intelligence and machine learning for plasmonic and surface-enhanced sensing. Chem. Soc. Rev..

[75] Yin B., Zhang Q., Xia X., Li C., Ho W.K.H., Yan J., Huang Y., Wu H., Wang P., Yi C. (2022). A CRISPR-Cas12a integrated SERS nanoplatform with chimeric DNA/RNA hairpin guide for ultrasensitive nucleic acid detection. Theranostics.

[76] Zhou C., Zhang Y., Yang X., Zhao Z., Xia M., Wei C., Wu Q., Chang Z., Ma L., Yin L. (2025). A magnetic CRISPR/Cas12a-SERS nanobiosensor for amplification-free and ultrasensitive detection of norovirus in water and food samples. Anal. Chim. Acta.

[77] Kissell L.N., Han D., Vang D., Cikanek A.W.R., Steckl A.J., Strobbia P. (2024). Improved point-of-care detection of *P. gingivalis* using optimized surface-enhanced Raman scattering in lateral flow assays. Sens. Diagn..

[78] Ho K.H.W., Lai H., Zhang R., Chen H., Yin W., Yan X., Xiao S., Lam C.Y.K., Gu Y., Yan J. (2024). SERS-Based Droplet Microfluidic Platform for Sensitive and High-Throughput Detection of Cancer Exosomes. ACS Sens..

[79] Yang J., Cui Y., Wang M., Zheng S., Luo S., Huang Y., He J., Zhang R., Jiang X., Zhong S. (2023). Application of serum SERS technology combined with deep learning algorithm in the rapid diagnosis of immune diseases and chronic kidney disease. Sci. Rep..

[80] Ho C.-S., Jean N., Hogan C.A., Blackmon L., Jeffrey S.S., Holodniy M., Banaei N., Saleh A.A.E., Ermon S., Dionne J. (2019). Rapid identification of pathogenic bacteria using Raman spectroscopy and deep learning. Nat. Commun..

[81] Tseng Y.-M., Chen K.-L., Chao P.-H., Han Y.-Y., Huang N.-T. (2023). Deep Learning–Assisted Surface-Enhanced Raman Scattering for Rapid Bacterial Identification. ACS Appl. Mater. Interfaces.

[82] Chen H., Zhao R., Bi X., Shen N., Mo X., Tao Y., Chen Z., Ye J. (2025). Bacterial identification by metabolite-level interpretable surface-enhanced Raman spectroscopy (SERSome). Adv. Photonics.

[83] Szymborski T.R., Berus S.M., Nowicka A.B., Słowiński G., Kamińska A. (2024). Machine Learning for COVID-19 Determination Using Surface-Enhanced Raman Spectroscopy. Biomedicines.

[84] Nyamdavaa A., Kaladharan K., Ganbold E.-O., Jeong S., Paek S., Su Y., Tseng F.-G., Ishdorj T.-O. (2025). DeepATsers: A deep learning framework for one-pot SERS biosensor to detect SARS-CoV-2 virus. Sci. Rep..

[85] Yuan R., Zhan H., Liu Y., Su J., Ju J., Qiao X. (2025). Integrating AI-assisted SERS Biosensing and Photoactivated Antibacterial Therapy in Au@Cu_2−x_Se for Combating Multidrug-Resistant Bacteria. Anal. Chem..

[86] Hu J., Chen G.J., Xue C., Shum P.P. (2024). RSPSSL: A novel high-fidelity Raman spectral preprocessing scheme to enhance biomedical applications and chemical resolution visualization. Light Sci. Appl..

[87] Zhao F., Zheng Y., Zhao Z., Wang W., Xu T., Xue X., Fu W., Ling Y., Shi J., Zhang Z. (2024). Re-understanding of SERS for General and Standardized Quantitative Analysis. Nano Lett..

[88] Cui J., Chen X., Zhao Y. (2025). Beyond Traditional airPLS: Improved Baseline Removal in SERS with Parameter-Focused Optimization and Prediction. Anal. Chem..

[89] Farnesi E., Guliev R., Liu C., Ballmaier J., Guntinas-Lichius O., Schmitt M., Popp J., Cialla-May D. (2025). Point-of-care SERS-based ML diagnosis of head and neck cancer via cerumen analysis. npj Biosens..

[90] Chen X., Wu X., Chen C., Luo C., Shi Y., Li Z., Lv X., Chen C., Su J., Wu L. (2023). Raman spectroscopy combined with a support vector machine algorithm as a diagnostic technique for primary Sjögren’s syndrome. Sci. Rep..

[91] Liu J., Osadchy M., Ashton L., Foster M., Solomon C.J., Gibson S.J. (2017). Deep convolutional neural networks for Raman spectrum recognition: A unified solution. Analyst.

[92] Wu M., Wang S., Pan S., Terentis A.C., Strasswimmer J., Zhu X. (2021). Deep learning data augmentation for Raman spectroscopy cancer tissue classification. Sci. Rep..

[93] Zaki J.K., Tomasik J., McCune J.A., Bahn S., Lió P., Scherman O.A. (2025). Explainable Deep Learning Framework for SERS Bioquantification. ACS Sens..

[94] Sounderajah V., Guni A., Liu X., Collins G.S., Karthikesalingam A., Markar S.R., Golub R.M., Denniston A.K., Shetty S., Moher D. (2025). The STARD-AI Reporting Guideline for Diagnostic Accuracy Studies Using Artificial Intelligence. Nat. Med..

[95] Ebrahimi F., Kumari A., Dellinger K. (2024). Integration of Nanoengineering with Artificial Intelligence and Machine Learning in Surface-Enhanced Raman Spectroscopy (SERS) for the Development of Advanced Biosensing Platforms. Adv. Sens. Res..

[96] Giordano A.N., Franqui-Rios S., Quarin S.M., Vang D., Austin D.R., Doyle A.G., Baldwin L.A., Strobbia P., Rao R. (2025). Fabrication of SERS Substrates Using Silver-Coated Gold Nanostars for Chemical Sensing: A Multiobjective Bayesian Optimization Approach. ACS Appl. Nano Mater..

[97] Liu A., Gao C., Xue J., Xie Y., Miao X., Chen T., Wu A., Lin J. (2026). Toward reproducible SERS biosensing: Analysing instability origins and mitigation approaches from sample preparation to detection. J. Mater. Chem. B.

[98] Lyu N., Hassanzadeh-Barforoushi A., Rey Gomez L.M., Zhang W., Wang Y. (2024). SERS biosensors for liquid biopsy towards cancer diagnosis by detection of various circulating biomarkers: Current progress and perspectives. Nano Converg..

[99] Zong C., Xu M., Xu L.-J., Wei T., Ma X., Zheng X.-S., Hu R., Ren B. (2018). Surface-Enhanced Raman Spectroscopy for Bioanalysis: Reliability and Challenges. Chem. Rev..

[100] Bratchenko I.A., Bratchenko L.A., Khristoforova Y.A., Moryatov A.A., Kozlov S.V., Zakharov V.P. (2022). Classification of skin cancer using convolutional neural networks analysis of Raman spectra. Comput. Methods Programs Biomed..

[101] Vázquez-Iglesias L., Stanfoca Casagrande G.M., García-Lojo D., Ferro Leal L., Ngo T.A., Pérez-Juste J., Reis R.M., Kant K., Pastoriza-Santos I. (2024). SERS sensing for cancer biomarker: Approaches and directions. Bioact. Mater..

[102] Zhao Z., Huang C., Zeng H. (2024). Zwitterion-Conjugated Protein Coatings for Enhanced Antifouling in Complex Biofluids: Underlying Molecular Interaction Mechanisms. Langmuir.

[103] Tan E.X., Nguyen L.B.T., Jin Y., Lv Y., Phang I.Y., Ling X.Y. (2025). SERS Cheminformatics: Opportunities for Data-Driven Discovery and Applications. ACS Cent. Sci..

[104] Tripathy S., Chavva S., Coté G.L., Mabbott S. (2023). Modular and handheld Raman systems for SERS-based point-of-care diagnostics. Curr. Opin. Biomed. Eng..

[105] Lian S., Li X., Lv X. (2025). Recent Developments in SERS Microfluidic Chips: From Fundamentals to Biosensing Applications. ACS Appl. Mater. Interfaces.

[106] Wang N., Weng Y., Liu Y., Wu Y., Weng S., Shen Y., Feng S., Lin D. (2025). Wearable nanoplasmonic sensor based on surface-enhanced Raman scattering for multiplexed analysis of sweat. Photon. Res..

[107] Duffield C., Rey Gomez L.M., Tsao S.C.-H., Wang Y. (2025). Recent advances in SERS assays for detection of multiple extracellular vesicles biomarkers for cancer diagnosis. Nanoscale.

[108] U.S. Food and Drug Administration (2021). Artificial Intelligence and Machine Learning (AI/ML) Software as a Medical Device Action Plan.

[109] Reddy S. (2025). Global Harmonization of Artificial Intelligence-Enabled Software as a Medical Device Regulation: Addressing Challenges and Unifying Standards. Mayo Clin. Proc. Digit. Health.

